# Web Impingement Syndrome of the Ankle: A Rare Cause of Persistent Post-traumatic Pain

**DOI:** 10.7759/cureus.97012

**Published:** 2025-11-16

**Authors:** Anis Soualili, Adel Arfi, Hamza Retal, Mohamed Khalil Khabet, Redouane Kadi

**Affiliations:** 1 Radiology, Helora University Hospital, Nivelles, BEL; 2 Radiology, Erasmus Hospital-Brussels, Brussels, BEL

**Keywords:** ankle arthroscopy, ankle impingement, chronic ankle pain, fibrous band, post-traumatic pain, talocrural joint, web impingement syndrome

## Abstract

Web impingement syndrome of the ankle is a rare and often underdiagnosed cause of persistent post-traumatic pain, mainly due to limited awareness of its clinical and radiological presentation. We report the case of a 54-year-old woman who presented with chronic ankle pain and limited mobility after a traumatic injury. Despite favorable healing of the osseous and ligamentous injuries on imaging, she continued to experience significant pain and functional limitation seven months later, necessitating further diagnostic workup. Clinical evaluation revealed localized tenderness over the anterior aspect of the ankle, along with a restricted range of motion, particularly in dorsiflexion. Imaging studies, including MRI, demonstrated joint effusion and identified an intra-articular fibrous band within the anterior talocrural recess, causing mechanical impingement and contributing to the patient’s symptoms. The patient underwent arthroscopic resection of the fibrous tissue, which resulted in gradual and sustained symptomatic improvement, with restoration of function during follow-up. This case highlights the importance of considering web impingement syndrome in the differential diagnosis of persistent post-traumatic ankle pain, particularly when standard imaging studies are inconclusive. Early recognition of this condition is essential to prevent chronic functional impairment and guide appropriate management. Arthroscopic intervention offers a minimally invasive and effective treatment option, leading to significant symptomatic relief and improved joint mobility. Increased awareness among orthopedic surgeons and radiologists can facilitate timely diagnosis and optimize patient outcomes in similar cases.

## Introduction

Ankle joint impingements represent a group of pathologies that occur either following trauma or as a result of repetitive microtrauma. They are characterized by localized mechanical pain and restricted range of motion, typically caused by the intra-articular interposition of osseous or soft tissue structures such as synovial hypertrophy, fibrous adhesions, or ossifications [1]. While anterior, anterolateral, anteromedial, posterior, and posteromedial impingement syndromes have been extensively described [2], less common and more recently recognized variants continue to emerge. Among these is web impingement syndrome, a recently described and underdiagnosed cause of persistent post-traumatic ankle pain. This condition involves the formation of fibrous bands or adhesions traversing the anterior recess of the tibiotalar joint [3]. These bands, often undetectable in the acute post-injury phase, are thought to result from organized hemarthrosis, particularly in the presence of significant joint effusion or intra-articular fracture [4].

This article aims to describe the clinical and imaging features of web impingement and emphasize the importance of recognizing this entity to ensure accurate diagnosis and effective treatment.

## Case presentation

**Figure 1:**
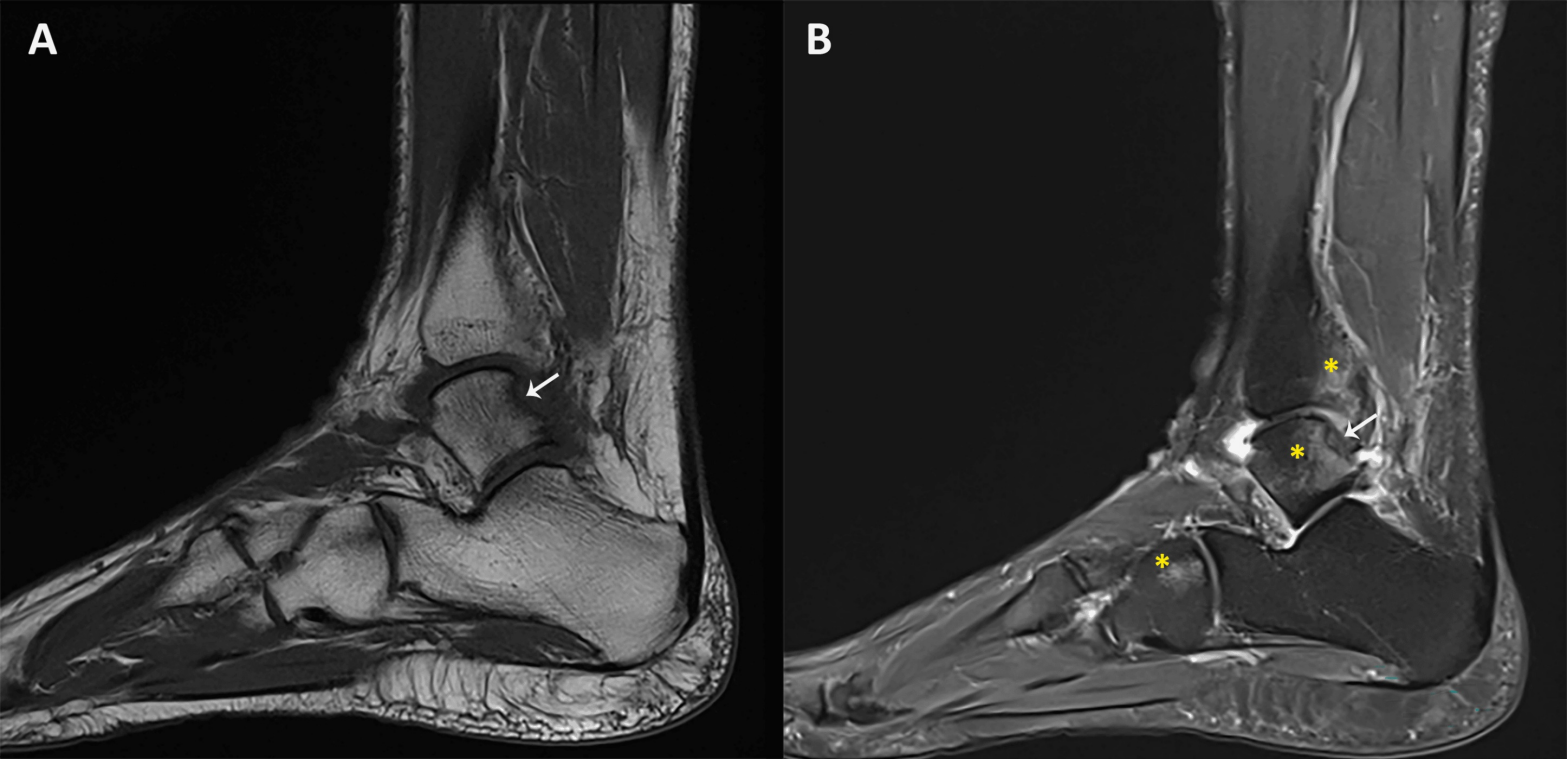
Sagittal MRI views of the ankle demonstrating a subchondral fracture and associated bone contusion. (A) T1-weighted image showing a subchondral fracture of the posterosuperolateral aspect of the talar dome (white arrow). (B) Proton density fat-saturated image highlighting bone contusion edema in the talus, cuboid, and distal tibia (yellow asterisk).

**Figure 2:**
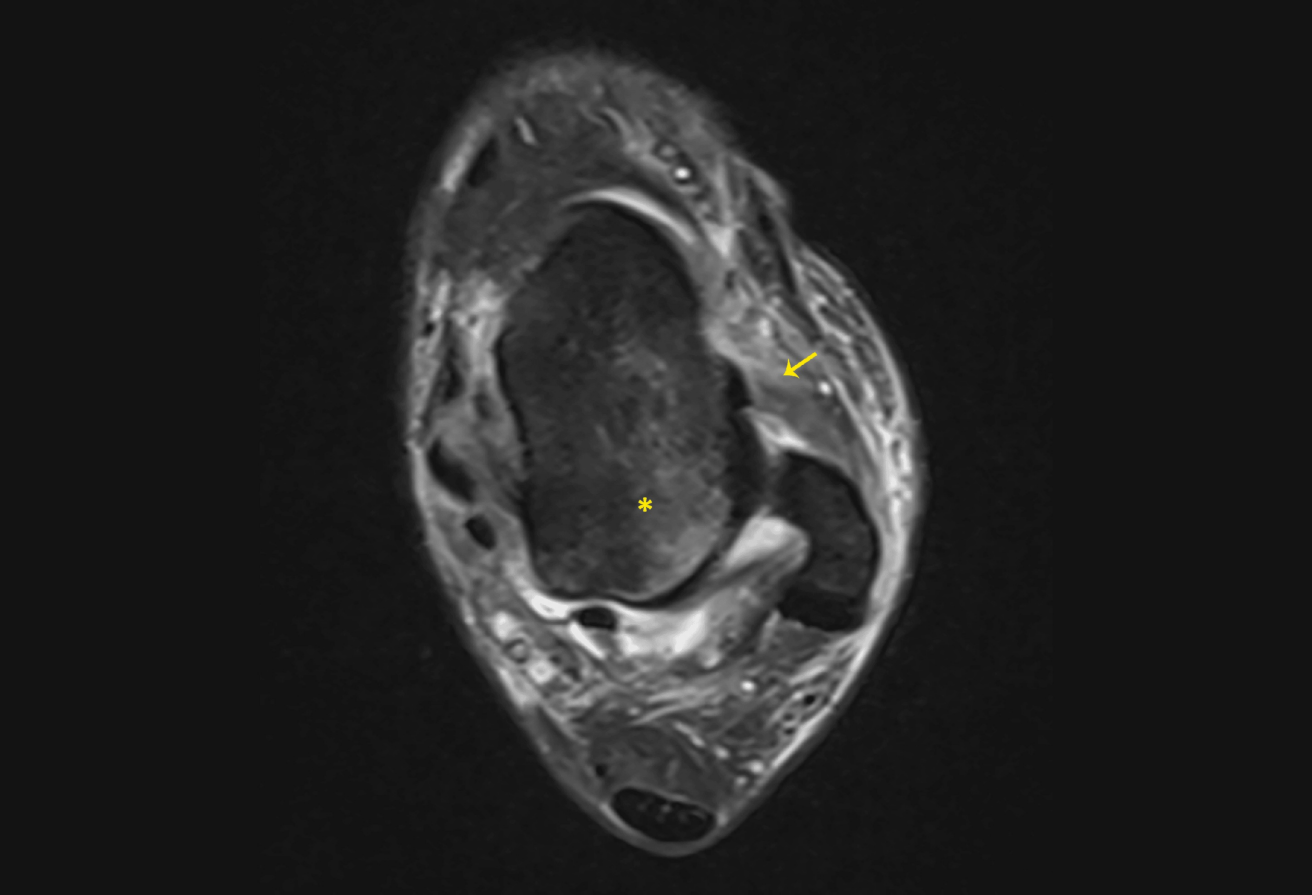
Axial proton density fat-saturated MRI of the ankle demonstrating soft tissue and bone injuries. The anterior talofibular ligament appears thickened and partially disrupted, consistent with a sprain (yellow arrow). Associated bone contusion edema is visible in the adjacent talus (yellow asterisk).

A 54-year-old woman with no significant medical history presented with left ankle pain and functional impairment following an inversion injury sustained after stepping into a hole. One month after the trauma, due to persistent pain and limited mobility, an MRI of the left ankle was performed. The MRI revealed a subchondral fracture involving the posterosuperolateral aspect of the talar dome, associated with bone contusion edema in the talus, cuboid, and lateral aspect of the distal tibia (Figure 1). It also showed an anterior talofibular ligament (ATFL) sprain (Figure 2).

**Figure 3:**
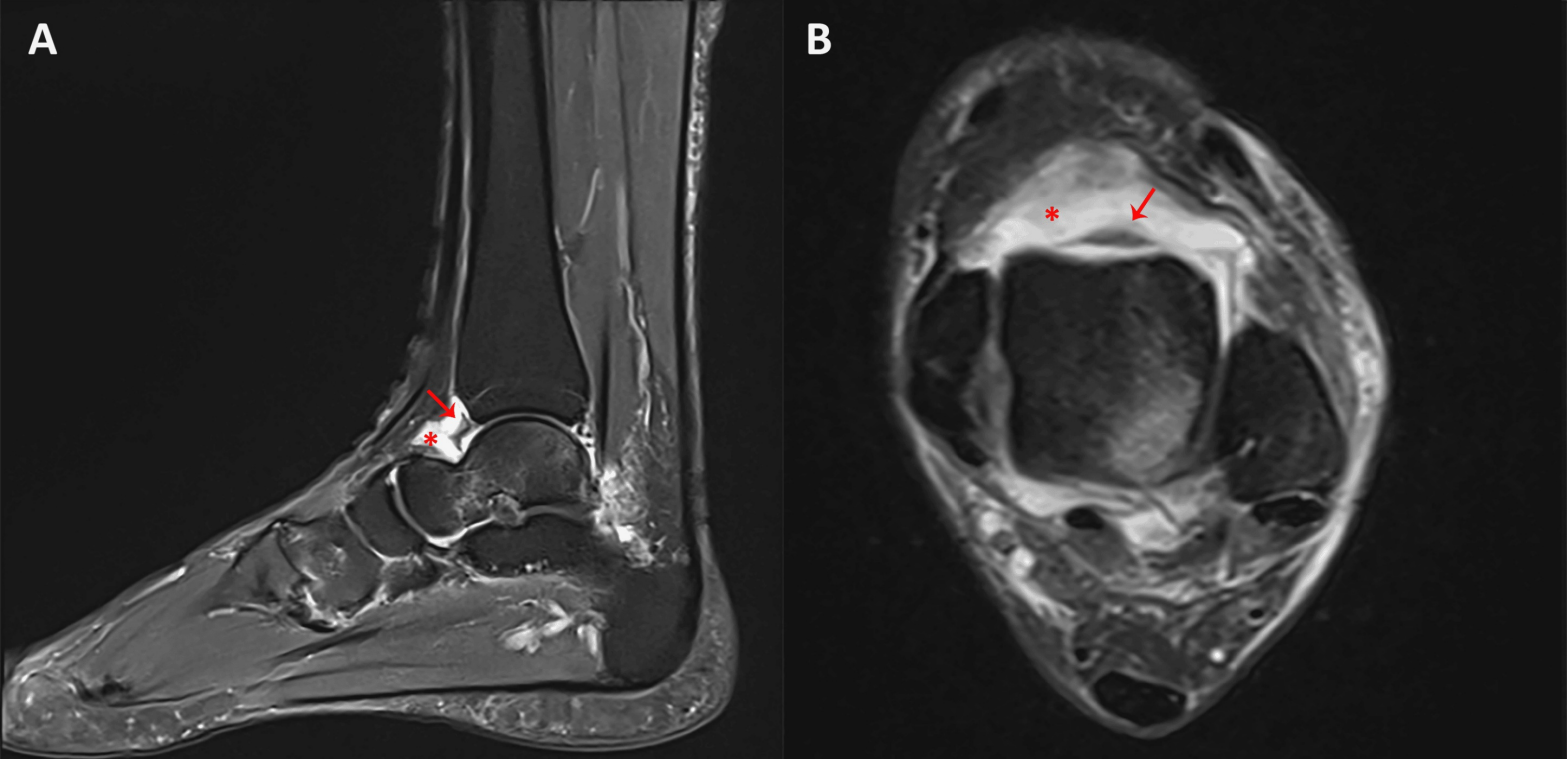
Sagittal and axial proton density fat-saturated MRI demonstrating intra-articular effusion and fibrous band formation. (A) Sagittal view showing a linear fibrous band traversing the anterior talocrural recess (red arrow) and associated joint effusion (red asterisk). (B) Axial view confirming the presence of the fibrous band (red arrow) and intra-articular fluid accumulation (red asterisk).

Additionally, a joint effusion was noted within the anterior talocrural recess, where a linear fibrous band was observed traversing the joint space (Figure 3).

The initial diagnosis included a subchondral fracture and ligamentous injury. A conservative treatment approach was initiated, including immobilization with an ankle brace and physical therapy. However, due to persistent symptoms, the patient was re-evaluated by an orthopedic specialist approximately seven months after the initial trauma.

**Figure 4:**
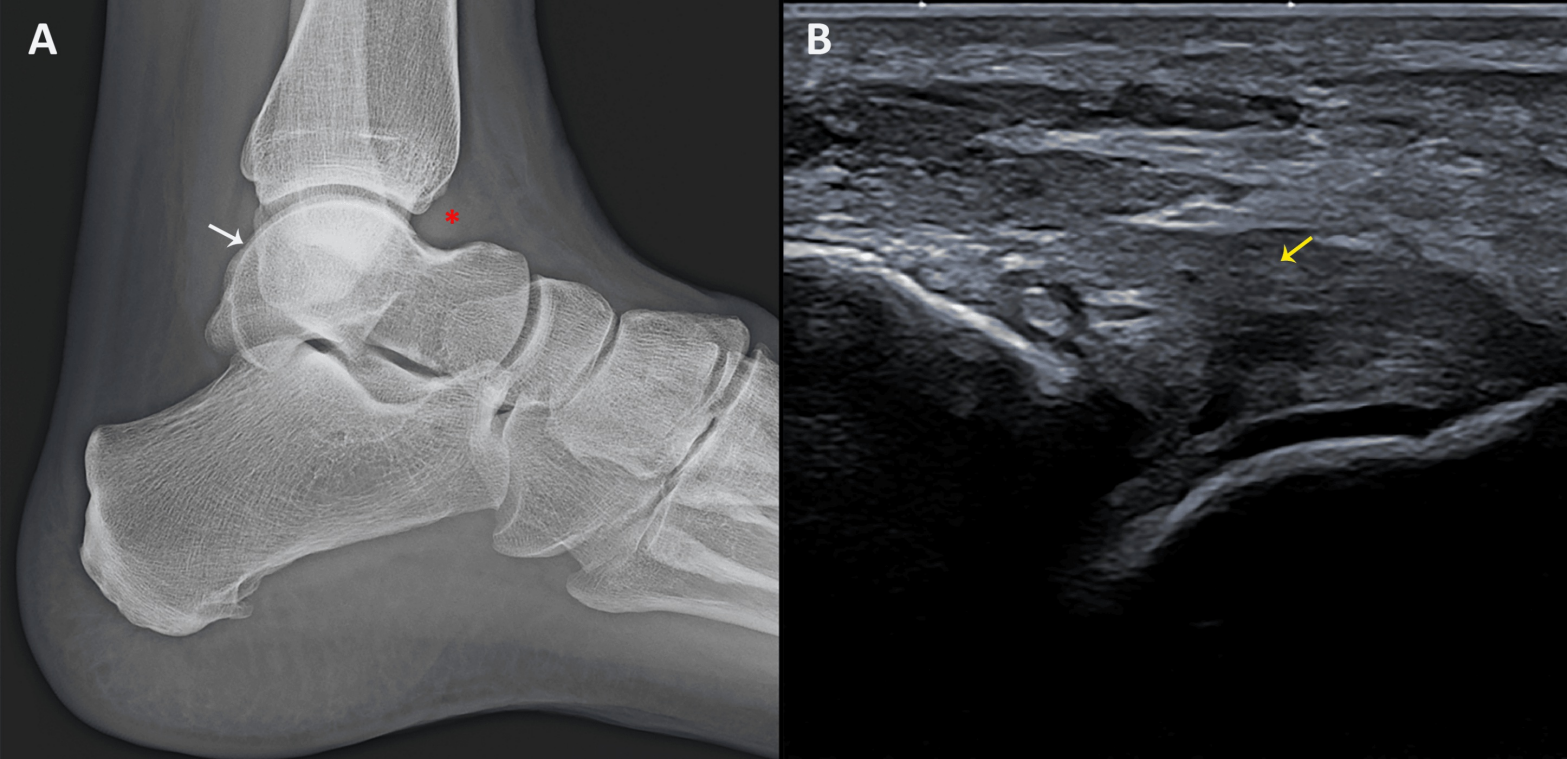
Lateral ankle radiograph and ultrasound image obtained during follow-up evaluation. (A) Lateral radiograph demonstrating satisfactory healing of the subchondral fracture involving the posterosuperolateral aspect of the talar dome (white arrow), as well as persistent joint effusion in the anterior talocrural recess (red asterisk). (B) Longitudinal ultrasound view of the anterior talofibular ligament showing ligamentous thickening consistent with post-sprain sequelae (yellow arrow).

At follow-up, clinical examination revealed localized pain in the anterior ankle joint and mechanical symptoms such as catching and discomfort during forced dorsiflexion, findings suggestive of soft tissue impingement, including possible web impingement. A plain radiograph demonstrated satisfactory healing of the subchondral talar fracture, while an ankle ultrasound showed thickening of the ATFL consistent with post-sprain sequelae (Figure 4).

**Figure 5:**
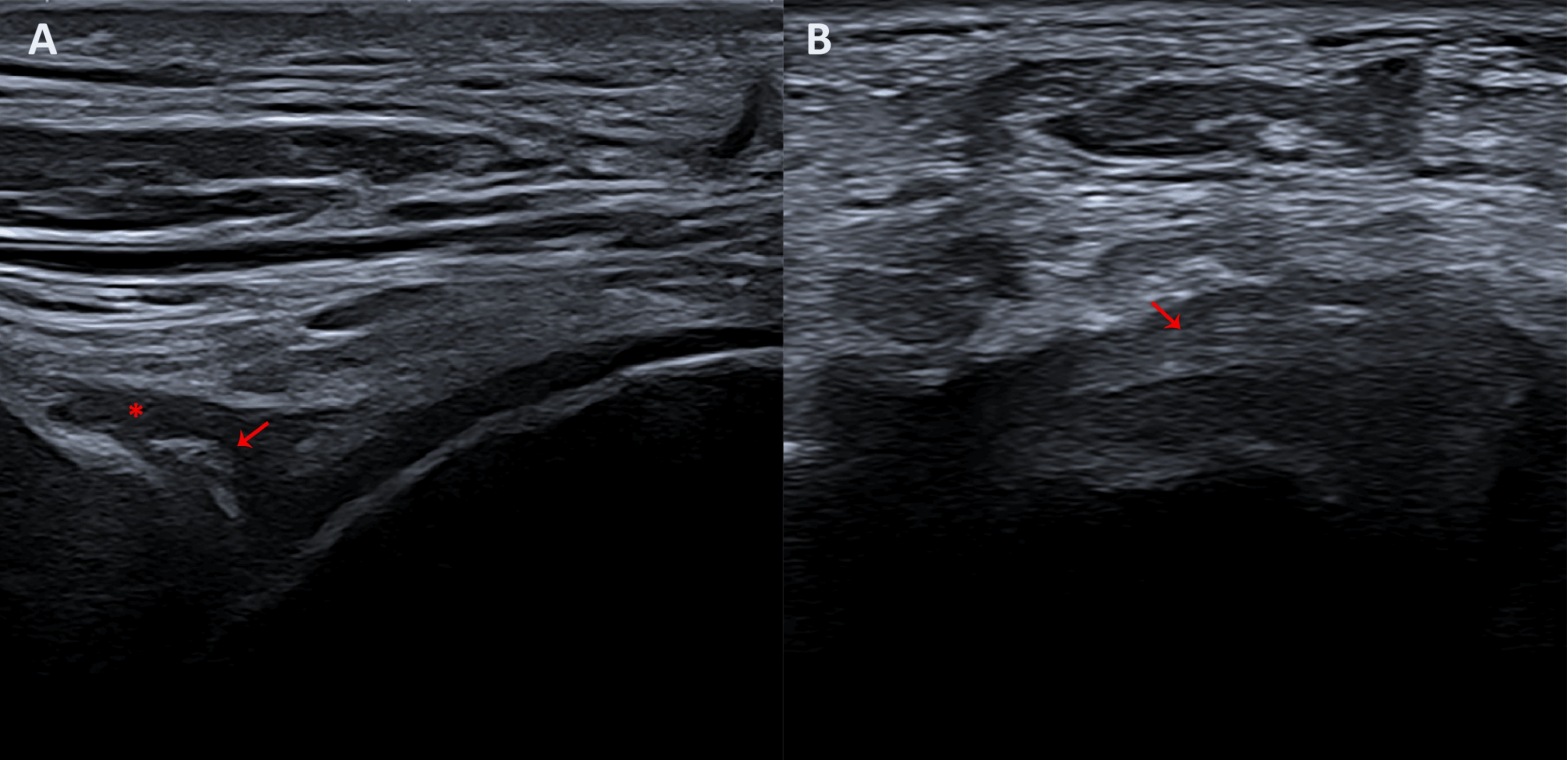
Ultrasound images focused on the anterior talocrural recess, confirming persistent intra-articular findings. (A) Longitudinal view demonstrating persistent joint effusion (red asterisk) and an intra-articular fibrous band traversing the recess (red arrow). (B) Axial view confirming the presence of the fibrous band (red arrow) within the anterior recess.

The ultrasound also revealed a persistent intra-articular effusion in the anterior talocrural recess. Notably, the fibrous band previously seen on MRI was again visualized (Figure 5).

**Figure 6:**
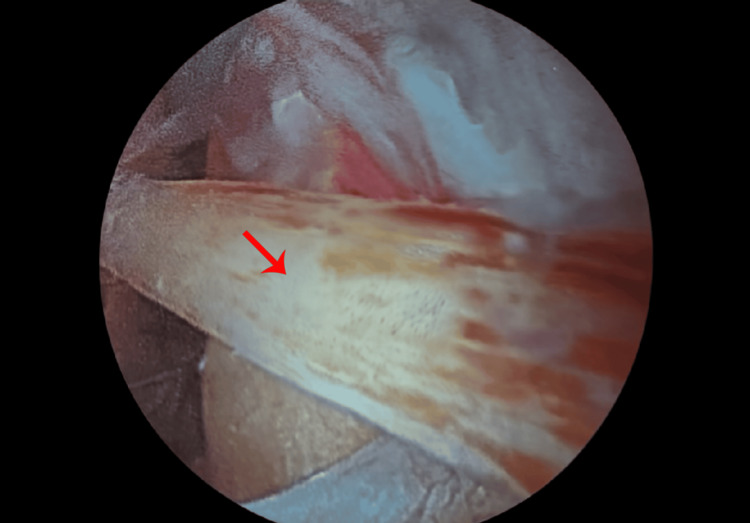
Arthroscopic image of the ankle demonstrating a fibrous band spanning the anterior talocrural recess (red arrow), responsible for mechanical impingement.

Based on the combination of persistent anterior ankle symptoms, imaging findings, and failure of conservative management, a diagnosis of web impingement syndrome was retained. The patient was referred to an orthopedic specialist for evaluation, who subsequently indicated ankle arthroscopy. During arthroscopy, a fibrous band spanning the anterior talocrural recess was clearly visualized and confirmed to be causing mechanical impingement (Figure 6).

The band was resected using standard instrumentation under direct visualization. The procedure was well tolerated, and postoperative recovery was uneventful. At follow-up, the patient reported progressive improvement in pain and ankle mobility, with complete resolution of mechanical symptoms.

## Discussion

Web impingement syndrome of the ankle is an uncommon and frequently underrecognized cause of persistent post-traumatic ankle pain. It is characterized by intra-articular fibrous bands or adhesions crossing the anterior recess of the tibiotalar joint, often resulting from the sequelae of trauma [3,5]. These fibrous bands can produce mechanical impingement, leading to localized pain and restriction of dorsiflexion and overall ankle mobility, as illustrated in our case.

The injury pattern observed in our patient, including a subchondral fracture of the posterosuperolateral talar dome, talar bone contusion, and ATFL tear, is consistent with a well-established complex inversion trauma model. Acute inversion ankle sprains most commonly involve injury to the ATFL, often accompanied by osseous lesions such as bone contusions or subchondral fractures. These osseous lesions frequently coexist with ligamentous injuries, particularly in high-grade sprains [6,7]. This combination may predispose patients to secondary complications such as soft tissue impingements, including web impingement syndrome.

The pathophysiology underlying web impingement is believed to involve the organization and fibrous transformation of intra-articular hemarthrosis following trauma [4]. Fibrous bands, rich in collagen and composed of normal fibroblasts, form within the anterior talocrural recess, an area prone to blood accumulation due to its volume. These fibrous structures are absent in the acute phase but develop over time, explaining the delayed onset of mechanical symptoms typical of web impingement [3,5]. Their transverse orientation across the joint recess further supports this mechanism [3].

Although epidemiological data remain limited, a retrospective series of 212 anterior ankle arthroscopies identified intra-articular fibrous bands in 19 (8.96%) patients, including six (2.83%) cases with isolated web-like adhesions without associated pathology. Notably, all of these patients were symptomatic, and arthroscopic resection led to significant clinical improvement, with the mean American Orthopaedic Foot and Ankle Society (AOFAS) ankle-hindfoot score increasing from 55.6 preoperatively to 92.3 postoperatively [5]. The AOFAS score is a standardized clinical scale that assesses pain, function, and alignment of the foot and ankle, with higher scores indicating better outcomes.

Clinically, web impingement presents as chronic anterior ankle pain; mechanical symptoms such as catching, clicking, or locking; and restricted range of motion, most notably in dorsiflexion [3,5]. Unlike classical anterolateral or anteromedial impingement syndromes, web impingement typically lacks characteristic bony spurs or osteophytic changes on plain radiographs, which may contribute to underdiagnosis [1,2].

The main differential diagnoses include other causes of anterior ankle impingement, such as anterolateral or anteromedial soft tissue impingement, hypertrophic synovitis, meniscoid lesions, and intra-articular loose bodies. Osseous impingement due to anterior tibial or talar osteophytes should also be considered. Additionally, chronic anterior ankle pain may result from post-traumatic chondral lesions or early post-traumatic osteoarthritis, which can mimic impingement symptoms. Accurate diagnosis relies on correlating clinical findings with imaging features, particularly on MRI, which can distinguish fibrous bands from these alternative entities.

Imaging is crucial for diagnosis. MRI is particularly useful, especially in the presence of joint effusion, which enhances visualization of fibrous bands as low-signal, linear structures traversing the anterior recess [6]. CT arthrography can also aid diagnosis, but is less commonly used. Ultrasound remains limited in detecting these bands due to their small size and variable morphology.

Arthroscopic intervention permits direct visualization and resection of fibrous bands, generally resulting in significant symptomatic relief and functional recovery [5,8]. Beaudet et al. reported four cases treated arthroscopically with excellent outcomes and no recurrence at follow-up, confirming the efficacy and safety of this minimally invasive approach for managing intra-articular fibrous bands [8]. Similarly, Cesaroni et al.’s retrospective series found that isolated web-like adhesions, though rare, responded favorably to arthroscopic removal, with substantial improvement in patient-reported outcomes [5]. Our patient’s clinical course aligns with these findings.

Given the low awareness and subtle clinical and imaging features, web impingement syndrome remains underdiagnosed. Awareness among orthopedic surgeons and radiologists should be increased, especially in patients with persistent post-traumatic ankle pain and limited dorsiflexion despite apparent healing on standard imaging. Early diagnosis and timely arthroscopic management are key to preventing chronic impairment.

## Conclusions

Web impingement syndrome of the ankle is a rare but important cause of persistent post-traumatic ankle pain, often overlooked due to subtle signs and limited awareness. Recognition is essential, especially in patients with unresolved symptoms despite healing of osseous and ligamentous injuries. MRI is valuable diagnostically, and arthroscopic resection of intra-articular fibrous bands remains the gold standard treatment, providing significant symptom relief and functional recovery. Increased vigilance by orthopedic surgeons and radiologists will improve timely diagnosis and optimize outcomes.
